# Health Utilities in People with Hepatitis C Virus Infection: A Study Using Real-World Population-Level Data

**DOI:** 10.1177/0272989X251319342

**Published:** 2025-02-22

**Authors:** Yasmin A. Saeed, Nicholas Mitsakakis, Jordan J. Feld, Murray D. Krahn, Jeffrey C. Kwong, William W. L. Wong

**Affiliations:** Leslie Dan Faculty of Pharmacy, University of Toronto, Toronto, ON, Canada; Toronto Health Economics and Technology Assessment (THETA) Collaborative, University Health Network, Toronto, ON, Canada; ICES, Toronto, ON, Canada; School of Pharmacy, University of Waterloo, Kitchener, Ontario, Canada; Children’s Hospital of Eastern Ontario Research Institute, Ottawa, ON, Canada; Toronto Centre for Liver Disease, University Health Network, Toronto, ON, Canada; Leslie Dan Faculty of Pharmacy, University of Toronto, Toronto, ON, Canada; Toronto Health Economics and Technology Assessment (THETA) Collaborative, University Health Network, Toronto, ON, Canada; ICES, Toronto, ON, Canada; ICES, Toronto, ON, Canada; Public Health Ontario, Toronto, ON, Canada; Dalla Lana School of Public Health, University of Toronto, Toronto, ON, Canada; Centre for Vaccine Preventable Diseases, University of Toronto, Toronto, ON, Canada; Department of Family and Community Medicine, University of Toronto, Toronto, ON, Canada; University Health Network, Toronto, ON, Canada; Leslie Dan Faculty of Pharmacy, University of Toronto, Toronto, ON, Canada; Toronto Health Economics and Technology Assessment (THETA) Collaborative, University Health Network, Toronto, ON, Canada; ICES, Toronto, ON, Canada; School of Pharmacy, University of Waterloo, Kitchener, Ontario, Canada

**Keywords:** hepatitis C, viral hepatitis, health utilities, patient preferences, health-related quality of life, patient reported outcome measures, quality-adjusted life years, cost-effectiveness analysis

## Abstract

**Background:**

Hepatitis C virus (HCV) infection is associated with reduced quality of life and health utility. It is unclear whether this is primarily due to HCV infection itself or commonly co-occurring patient characteristics such as low income and mental health issues. This study aims to estimate and separate the effects of HCV infection on health utility from the effects of clinical and sociodemographic factors using real-world population-level data.

**Methods:**

We conducted a cross-sectional retrospective cohort study to estimate health utilities in people with and without HCV infection in Ontario, Canada, from 2000 to 2014 using linked survey data from the Canadian Community Health Survey and health administrative data. Utilities were derived from the Health Utilities Index Mark 3 instrument. We used propensity score matching and multivariable linear regression to examine the impact of HCV infection on utility scores while adjusting for clinical and sociodemographic factors.

**Results:**

There were 7,102 individuals with hepatitis C status and health utility data available (506 HCV-positive, 6,596 HCV-negative). Factors associated with marginalization were more prevalent in the HCV-positive cohort (e.g., household income <$20,000: 36% versus 15%). Propensity score matching resulted in 454 matched pairs of HCV-positive and HCV-negative individuals. HCV-positive individuals had substantially lower unadjusted utilities than HCV-negative individuals did (mean ± standard error: 0.662 ± 0.016 versus 0.734 ± 0.015). The regression model showed that HCV positivity (coefficient: −0.066), age, comorbidity, mental health history, and household income had large impacts on health utility.

**Conclusions:**

HCV infection is associated with low health utility even after controlling for clinical and sociodemographic variables. Individuals with HCV infection may benefit from additional social services and supports alongside antiviral therapy to improve their quality of life.

**Highlights:**

- Hepatitis C virus (HCV) infection is associated with reduced quality of life and health utility. There is debate in the literature on whether this is primarily due to HCV infection itself or commonly co-occurring patient characteristics such as low income and mental health issues.
- We showed that individuals with HCV infection have substantially lower health utilities than uninfected individuals do even after controlling for clinical and sociodemographic variables, based on a large, real-world population-level dataset. Socioeconomically marginalized individuals with HCV infection had particularly low health utilities.
- In addition to improving access to HCV testing and treatment, it may be beneficial to provide social services such as mental health and financial supports to improve the quality of life and health utility of people living with HCV.

## Introduction

Chronic hepatitis C virus (HCV) infection is estimated to affect 58 million people worldwide.^1^ This condition is associated with reduced quality of life^2^ and health utility.^3^ However, there is debate surrounding the health utility decrement associated with chronic hepatitis C. This debate revolves around the question of how much of these patients’ impairment in health utility is associated with HCV infection itself compared with commonly co-occurring patient characteristics including current or former substance use, homelessness or unstable housing, mental health issues, and other factors associated with low socioeconomic status.^4,5^

This is an important question that could guide models of care for HCV patients.^6^ If HCV infection is not a large determining factor in these patients’ health utility, then antiviral therapy alone will not be sufficient to improve their health utility. Exploring this question and its implications for resource allocation could lead to the development of additional programs to target social determinants of health and comorbid health conditions alongside HCV treatment.

Health utility is a global measure of health status and a useful outcome measure to guide medical decision making. Health utilities are anchored at 0 (equivalent to dead) and 1 (equivalent to perfect health). They incorporate not only the severity of a disease but also patient or societal preference for that health state and allow us to calculate quality-adjusted life-years. This is a meaningful method of measuring the burden of a disease in a way that goes beyond mortality. Health utilities and quality-adjusted life-years are useful for quantifying and comparing disease burden in individuals and populations, measuring the potential benefits and harms of clinical and policy interventions, and conducting cost-utility analysis to guide resource allocation.

Few studies on health utilities in HCV patients have included a comparable non–HCV-infected control group and adequate sample size to analyze multiple variables simultaneously to understand the effect of HCV on health utility and the causes of reduced health utility in HCV patients.^7–9^ These studies tend to rely on self-reported HCV infection status, which may be inaccurate. To our knowledge, this is the first study to compare health utilities in a large general population sample of hepatitis C patients and uninfected individuals based on HCV testing records. The primary objective of this study is to use population-level survey and health care administrative data to estimate the effect of HCV infection on health utility. Our secondary objective is to examine the effects of comorbid mental health issues, household income, and other clinical and sociodemographic factors on health utility.

## Methods

### Study Design

This is a retrospective cross-sectional study of health utilities in people with and without HCV infection using linked survey and health care administrative data from Ontario, Canada.

### Ethics

This study received ethics approval from the University of Toronto.

### Data Sources

This study linked health care usage data and health survey data housed at ICES (formerly the Institute for Clinical Evaluative Sciences) and hepatitis C testing data housed in the Public Health Ontario Laboratory (PHOL) database. ICES is an independent, nonprofit research institute whose legal status under Ontario’s health information privacy law allows it to collect and analyze health care and demographic data, without consent, for health system evaluation and improvement. Public Health Ontario performs almost all HCV ribonucleic acid (RNA) testing for Ontario as well as some serologic testing^10^ at its 11 laboratories across the province.^11^

Health utilities were obtained from the Health Utilities Index Mark 3 (HUI3) questionnaire administered as part of the Canadian Community Health Survey (CCHS). The CCHS is an annual cross-sectional population-based health survey administered by Statistics Canada to a different representative sample of Canadians aged 12 y and older each year.^12^ Some questionnaires differ from year to year while others are included every year. The HUI3 questionnaire was administered in the 2000–2001,^13^ 2009–2010,^14^ and 2013–2014^15,16^ cycles of the CCHS in Ontario.

The HUI3 is a multiattribute utility instrument consisting of questions relating to 8 domains of health (vision, hearing, speech, ambulation, dexterity, emotion, cognition, and pain) that can capture 972,000 unique health states.^17^ Health utility scores can be calculated based on a reference dataset of societal preferences for these health states, which have previously been obtained using the Standard Gamble instrument in a Canadian population.^18^

Additional information including rurality, self-reported household income, race, immigrant status, marital status, and other sociodemographic variables were also obtained from the CCHS.

CCHS data were linked to hepatitis C testing data from the PHOL database. In addition, linkage to the following Ontario health care administration databases housed at ICES was performed to identify liver disease severity and comorbid health conditions: the Ontario Health Insurance Plan (OHIP) Claims Database (for physician claims), Canadian Institute for Health Information (CIHI) Discharge Abstract Database (DAD) (for hospital inpatient stays), National Ambulatory Care Reporting System (NACRS) (for emergency department visits and some hospital-based outpatient care), and Ontario Drug Benefit (ODB) (for drug claims).^19^

Liver cirrhosis was coarsely assessed by the presence or absence of International Classification of Diseases, Ninth Revision (ICD-9) and Tenth Revision (ICD-10) codes associated with cirrhosis (Appendix 2).

Comorbidity was measured using the Johns Hopkins Adjusted Clinical Group (ACG)^®^ system (version 10)^20^ Resource Utilization Bands (RUBs). This system uses ICD-9 and ICD-10 codes (which are available in the databases mentioned above [OHIP, DAD, NACRS]) to classify comorbid conditions into 1 of 32 diagnosis clusters based on duration, severity, diagnostic certainty, etiology, and specialty care involvement. These clusters can be collapsed into RUBs ranging from 0 to 5, with higher RUBs indicating greater morbidity and expected health care utilization (RUB 0: nonusers; RUB 1: healthy users; RUB 2: low morbidity; RUB 3: moderate morbidity; RUB 4: high morbidity; RUB 5: very high morbidity). The ACG system has the advantage of incorporating both ambulatory and inpatient diagnostic codes.

The Registered Persons Database was used to obtain the date of birth and date of death (if applicable).

These datasets were linked using unique encoded identifiers and analyzed at ICES.

### Study Population

All Ontario residents who answered the HUI3 questionnaire in the 2000–2001, 2009–2010, or 2013–2014 cycle of the CCHS were eligible for inclusion. Eligible individuals had to have linked data available on their hepatitis C status from the PHOL database and had to be OHIP eligible (i.e., covered by the provincial government’s health insurance plan) for at least 1 y prior to completing the CCHS.

In the base-case analysis, the HCV-positive cohort was defined as those who ever tested positive for HCV infection (HCV RNA or antibody [Ab] positive), while the HCV-negative cohort was defined as those who were tested for HCV infection but never had a positive test (no positive HCV RNA and/or antibody tests). We chose to define the HCV-negative group as tested HCV-negative individuals rather than untested individuals with the aim of minimizing differences between groups aside from HCV infection status. Since HCV testing is often risk factor based, these groups are likely more similar than HCV-positive individuals and untested individuals in terms of both measured and unmeasured risk factors. Individuals who were HCV positive at some point but whose most recent RNA test before their CCHS survey date was negative (i.e., past infection) were excluded from the base-case analysis because they may be a distinct group with differing health utilities but had too small of a sample size to analyze separately.

Individuals with a missing/invalid health card number, age, or sex were excluded. Those who did not have linked data available were excluded. Individuals who responded to the CCHS in more than one of the included cycles had their most recent survey’s responses included.

### Propensity Score Matching

We used propensity score matching (PSM) to address confounding arising from differing patient characteristics in the study groups. This is done by calculating a propensity score for each individual using logistic regression, which in this case represents the probability of being HCV positive based on observed covariates. Each HCV-positive case is then matched to 1 HCV-negative control with a similar propensity score, which balances the distribution of these observed covariates between groups.^21^

PSM was done using the “MatchIt”^22^ package in R. Propensity scores were calculated based on the following variables: age, sex, marital status, immigrant status, race, rurality, household income, comorbidity (based on Johns Hopkins ACG System RUB), mental health history (history of inpatient or outpatient mental health visit), liver cirrhosis, and which CCHS cycle the individual’s utility score was obtained from. Nearest-neighbour matching (also known as greedy matching) was performed with a caliper width of 0.2 using the logit of the propensity score.^23^ Standardized mean differences in baseline characteristics between the HCV-positive and HCV-negative cohorts were compared before and after PSM. All analyses described below were performed on the propensity score–matched data except for the sensitivity analysis.

### Analysis

Patient characteristics of the HCV-positive group and HCV-negative group were compared before and after PSM, including age, sex, marital status, immigrant status, race, rurality, household income, comorbidity (RUB), mental health history, liver cirrhosis, and CCHS cycle. Unadjusted mean health utility scores were estimated and compared for the propensity score–matched HCV-positive group and HCV-negative group. Bivariate analysis was done to estimate mean health utilities for the PSM HCV–positive and HCV–negative cohorts while stratifying by the above-mentioned patient characteristics.

A multivariable linear regression was performed on the PSM data, with health utility as the dependent variable and the following as independent variables: HCV infection status (HCV positive or HCV negative), age (by decades), sex, marital status, immigrant status, race, rurality, household income, comorbidity (RUB), mental health history, liver cirrhosis, and CCHS cycle. The regression coefficient for HCV status indicates whether HCV itself has a substantial impact on health utility when the other clinical and sociodemographic variables are controlled for. The other variables provide information on which other factors have important effects on health utilities in those who have hepatitis C or have been tested for hepatitis C.

Health utility data typically violates some of the assumptions required for linear regression models. Alternative models such as beta regression, Tobit, and censored least absolute deviations (CLAD) have been explored as more appropriate alternatives. However, several studies comparing these models have concluded that linear regression with nonparametric bootstrapped confidence intervals (CIs) performs adequately for modeling health utility data.^24,25^ Therefore, to account for the skewed and heteroscedastic nature of health utility data, we estimated 95% CIs for the regression coefficients using a nonparametric bootstrap. We used the “Boot” function from the “car”^26^ package in R to run 10,000 bootstrap samples of the multivariable regression model. Then, we used the “confint” function to generate bias-corrected and accelerated 95% CIs, as this method corrects for bias and skewness in the distribution of bootstrap estimates.^25^

All analyses were performed in R (R Foundation for Statistical Computing, Vienna, Austria).^27^

### Sensitivity Analysis: Alternate Classifications of HCV Infection/Testing Status

We assessed the impact of alternate classifications of HCV infection status and HCV testing status on health utility using the full cohort of CCHS respondents (i.e., unmatched data). Utility scores were compared among the following subgroups: those who had at least 1 hepatitis C antibody or RNA test versus those never tested; among those tested, those who ever tested RNA or Ab positive versus those who never tested positive; among those ever HCV positive, those tested RNA positive versus those tested Ab positive with no positive RNA test or no RNA testing data; and among those who tested Ab positive but never RNA positive, those tested Ab positive RNA negative versus those tested Ab positive RNA inconclusive versus those tested Ab positive with no RNA testing data.

### Role of Funding Sources

The funding sources were not involved in the design, conduct, or writing of this study.

## Results

There were 97,147 CCHS respondents with HUI data available. Of these, 7,158 individuals had HCV testing records: 506 were HCV positive and 6,596 were HCV negative. (Fifty-six individuals were excluded from the base-case analysis because they were HCV positive at some point but their last RNA test before their CCHS survey date was negative.) PSM resulted in 454 matched pairs of HCV-positive and HCV-negative individuals.

Before PSM, the HCV-positive cohort was older (69% versus 52% aged ≥40 y) and more likely to be male (62% versus 41%), be separated or divorced (26% versus 13%), have a low household income (36% versus 15% <$20,000 annually), have a high level of comorbidity (21% versus 11% RUB = 5), have a history of mental health issues (37% versus 22%), have liver cirrhosis (14% versus 2%), and have a utility score from an earlier CCHS cycle (43% versus 34% 2000–2001 cycle) (Table 1). Standardized mean differences were substantially reduced after PSM (Appendix 1 Table A1.1).

**Table 1:** Patient Characteristics by Hepatitis C Virus Infection Status, before and after Propensity Score Matching

	Before Matching		After Matching	
	HCV− (*n* = 6,596)	HCV+ (*n* = 506)	HCV− (*n* = 454)	HCV+ (*n* = 454)
Age, y				
12–19	798 (12%)	30 (6%)	40 (9%)	25 (6%)
20–39	2,346 (36%)	129 (26%)	145 (32%)	115 (25%)
40–59	1,874 (28%)	266 (53%)	144 (32%)	242 (53%)
60–79	1,330 (20%)	74 (15%)	110 (24%)	65 (14%)
80 or older	248 (4%)	7 (1%)	15 (3%)	7 (2%)
Sex (male)	2,732 (41%)	312 (62%)	261 (57%)	276 (61%)
Marital status				
Single	2,261 (34%)	171 (34%)	152 (34%)	149 (33%)
Married or common-law	3,036 (46%)	183 (36%)	183 (40%)	169 (37%)
Separated or divorced	826 (13%)	131 (26%)	96 (21%)	116 (26%)
Widowed	467 (7%)	21 (4%)	23 (5%)	20 (4%)
Missing^a^	6 (0%)	0 (0%)	—	—
Immigrant status				
Immigrant	1,112 (17%)	65 (13%)	62 (14%)	57 (13%)
Missing^a^	19 (0%)	0 (0%)	—	—
Race				
White	5,643 (86%)	437 (86%)	388 (86%)	397 (87%)
Asian^b^	289 (4%)	8 (2%)	11 ( 2%)	8 (2%)
Other	520 (8%)	52 (10%)	55 (12%)	49 (11%)
Missing^a^	144 (2%)	9 (2%)	—	—
Rurality				
Rural	1,563 (24%)	92 (18%)	77 (17%)	83 (18%)
Missing^a^	7 (0%)	2 (0%)	—	—
Household income				
No income or <$20,000	965 (15%)	183 (36%)	173 (38%)	168 (37%)
$20,000–$39,999	1,221 (19%)	108 (21%)	95 (21%)	106 (23%)
$40,000–$59,999	1,097 (17%)	80 (16%)	80 (18%)	79 (17%)
$60,000–$79,999	898 (14%)	52 (10%)	48 (11%)	52 (12%)
$80,000 or more	1,955 (30%)	49 (10%)	58 (13%)	49 (11%)
Missing^a^	460 (7%)	34 (7%)	—	—
Resource utilization band^c^				
0 (Nonusers)	277 (4%)	16 (3%)	12 (3%)	13 (3%)
1 (Healthy users)	267 (4%)	13 (3%)	14 (3%)	12 (3%)
2 (Low morbidity)	886 (13%)	54 (11%)	52 (12%)	46 (10%)
3 (Moderate morbidity)	3,098 (47%)	200 (40%)	192 (42%)	187 (41%)
4 (High morbidity)	1,367 (21%)	115 (23%)	95 (21%)	103 (23%)
5 (Very high morbidity)	701 (11%)	108 (21%)	89 (20%)	93 (21%)
Mental health history^d^	1,442 (22%)	185 (37%)	160 (35%)	167 (37%)
Liver cirrhosis	159 (2%)	73 (14%)	70 (15%)	59 (13%)
CCHS cycle				
2000–2001	2,233 (34%)	218 (43%)	192 (42%)	199 (44%)
2009–2010	2,299 (35%)	164 (32%)	136 (30%)	135 (30%)
2013–2014	2,064 (31%)	124 (25%)	126 (28%)	120 (26%)

CCHS, Canadian Community Health Survey; HCV, hepatitis C virus. See Appendix 1 for standardized mean differences before and after propensity score matching.

a Number of missing values displayed for variables with missing values; all other variables had no missing values.

b Asian includes West Central Asian and Middle Eastern, South Asian, East and Southeast Asian, and “other” Asian origins; based on Statistics Canada categories.

c Johns Hopkins Adjusted Clinical Group^®^ (ACG) system.

d History of hospital or outpatient mental health visit(s).

The HCV-positive cohort had substantially lower unadjusted health utilities than the HCV-negative cohort did (0.662 versus 0.734; Table 2). Unadjusted health utilities were also lower for the HCV-positive cohort when stratifying by age, sex, marital status, immigrant status, race, rurality, household income, comorbidity, mental health history, liver cirrhosis, and CCHS cycle, with few exceptions (those of rural residence and those in the “healthy users” resource utilization band [RUB = 1]; Table 2).

**Table 2:** Health Utilities Stratified by Hepatitis C Virus Infection Status and Other Factors (Unadjusted Mean ± Standard Error)

	HCV− (*n* = 454)	HCV+ (*n* = 454)
(All)	0.734 ± 0.015	0.662 ± 0.016
Age, y		
12–19	0.868 ± 0.032	0.799 ± 0.054
20–39	0.793 ± 0.023	0.757 ± 0.027
40–59	0.699 ± 0.026	0.611 ± 0.022
60–79	0.670 ± 0.034	0.648 ± 0.042
80 or older	0.601 ± 0.104	0.495 ± 0.124
Sex		
Male	0.763 ± 0.018	0.644 ± 0.020
Female	0.694 ± 0.024	0.690 ± 0.025
Marital status		
Single	0.787 ± 0.023	0.665 ± 0.027
Married or common-law	0.736 ± 0.024	0.708 ± 0.024
Separated or divorced	0.618 ± 0.033	0.598 ± 0.033
Widowed	0.846 ± 0.050	0.612 ± 0.082
Immigrant status		
Not an immigrant	0.735 ± 0.015	0.659 ± 0.017
Immigrant	0.728 ± 0.045	0.680 ± 0.044
Race		
White	0.739 ± 0.016	0.667 ± 0.016
Asian^a^	0.735 ± 0.105	0.728 ± 0.128
Other	0.696 ± 0.043	0.605 ± 0.055
Rurality		
Not rural	0.748 ± 0.016	0.660 ± 0.017
Rural	0.663 ± 0.040	0.669 ± 0.037
Household income		
No income or <$20,000	0.642 ± 0.026	0.520 ± 0.027
$20,000–$39,999	0.703 ± 0.036	0.669 ± 0.031
$40,000–$59,999	0.797 ± 0.028	0.795 ± 0.028
$60,000–$79,999	0.840 ± 0.030	0.816 ± 0.033
$80,000 or more	0.882 ± 0.026	0.754 ± 0.040
Resource utilization band^b^		
0 (Nonusers)	0.916 ± 0.038	0.847 ± 0.042
1 (Healthy users)	0.795 ± 0.071	0.887 ± 0.043
2 (Low morbidity)	0.917 ± 0.019	0.776 ± 0.044
3 (Moderate morbidity)	0.790 ± 0.020	0.700 ± 0.024
4 (High morbidity)	0.653 ± 0.035	0.648 ± 0.031
5 (Very high morbidity)	0.557 ± 0.037	0.488 ± 0.036
Mental health history^c^		
No mental health history	0.779 ± 0.017	0.714 ± 0.019
Mental health history	0.650 ± 0.026	0.571 ± 0.026
Liver cirrhosis		
No cirrhosis	0.755 ± 0.015	0.674 ± 0.017
Cirrhosis	0.614 ± 0.045	0.580 ± 0.044
CCHS cycle		
2000–2001	0.771 ± 0.021	0.729 ± 0.022
2009–2010	0.751 ± 0.026	0.599 ± 0.030
2013–2014	0.658 ± 0.030	0.622 ± 0.030

CCHS, Canadian Community Health Survey; HCV, hepatitis C virus.

a Asian includes West Central Asian and Middle Eastern, South Asian, East and Southeast Asian, and “other” Asian origins; based on Statistics Canada categories.

b Johns Hopkins Adjusted Clinical Group^®^ (ACG) system.

c History of hospital or outpatient mental health visit(s).

**Figure 1:**
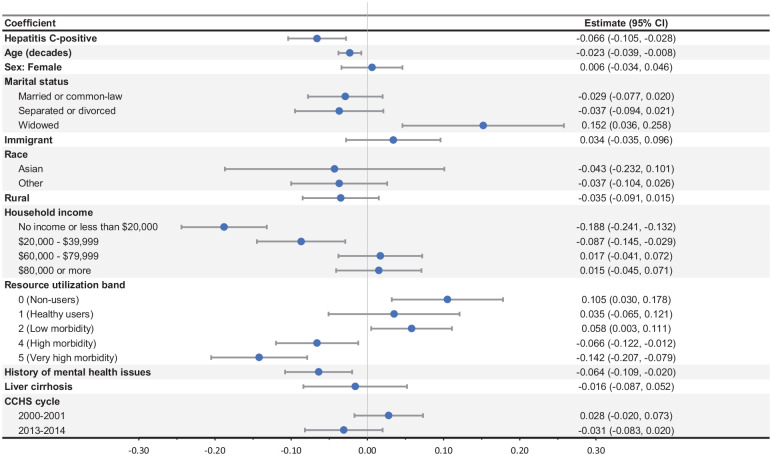
Results of regression analysis. Reference case: Hepatitis C infection status: hepatitis C negative; sex: male; marital status: single; immigrant status: not an immigrant; race: White; rurality: not rural; household income: $40,000–$59,999; Resource Utilization Band (a proxy for comorbidity): 3; mental health history: no history of mental health issues; liver cirrhosis status: no cirrhosis; CCHS cycle: 2009–2010. CCHS, Canadian Community Health Survey.

Multivariable regression analysis showed that HCV infection had a large and statistically significant negative effect on health utility (i.e., the 95% CI did not contain 0; coefficient [95% CI]: −0.066 [−0.105, −0.028]; Figure 1). The effects of age (−0.023 [−0.039, −0.008] per decade), having a low income (less than $20,000: −0.188 [−0.241, −0.132]; $20,000–$39,999: −0.087 [−0.145, −0.029]), having high comorbidity (RUB 4: −0.066 [−0.122, −0.012]; RUB 5: −0.142 [−0.207, −0.079]), and having a history of mental health issues (−0.064 [−0.109, −0.020]) were also large and statistically significantly associated with lower health utilities. Those who were widowed (0.152 [0.036, 0.258]) and those who had low comorbidity (RUB 0: 0.105 [0.030, 0.178]; RUB 2: 0.058 [0.003, 0.111]) had statistically significantly higher health utilities.

### Results of the Sensitivity Analysis

When comparing the HCV tested and untested cohorts, those never tested for HCV had higher health utilities (0.851 versus 0.802; Table 3). Among those tested, individuals who ever tested Ab or RNA positive had lower health utilities than those who never tested positive (0.658 versus 0.814). Among those tested who ever had a positive Ab or RNA test, those who ever tested RNA positive had higher utilities than those who ever tested Ab positive but never tested RNA positive (0.691 versus 0.597). Among those who ever tested Ab positive but never tested RNA positive, those who tested RNA negative had higher utilities than those who had no RNA testing data available (0.638 versus 0.581) or had an inconclusive RNA test result (0.566).

**Table 3:** Sensitivity Analysis of the Alternate Classification of Hepatitis C Virus Infection Status (Mean ± Standard Error)

HCV Status	*n*	Health Utility
Everyone	97,147	0.847 ± 0.001
Untested	89,989	0.851 ± 0.001
Tested (Ab and/or RNA tested)	7,158	0.802 ± 0.003
Tested, RNA and/or Ab negative	6,596	0.814 ± 0.003
Tested, ever RNA or Ab positive	562	0.658 ± 0.014
Ever RNA positive	362	0.691 ± 0.016
Ever Ab positive and never RNA positive	200	0.597 ± 0.025
Ab positive and RNA never tested	113	0.581 ± 0.034
Ab positive and RNA negative	62	0.638 ± 0.045
Ab positive and RNA inconclusive	25	0.566 ± 0.069

Ab, antibody; HCV, hepatitis C virus; RNA, ribonucleic acid.

## Discussion

Our results suggest that HCV infection is associated with reduced health utility and that this negative association remains after adjusting for clinical and sociodemographic factors. We found that age, low household income, high comorbidity, and a history of mental health issues were also important predictors of lower health utility. HCV infection was associated with a reduction of 0.066 in health utility, which is substantially greater than the commonly cited 0.03 threshold for clinical significance.^28,29^

This is a large effect relative to the symptoms typically associated with HCV infection, which is often asymptomatic for patients with mild disease—86% of our sample had mild disease (no cirrhosis). This reduced quality of life could be explained in part by nonspecific symptoms experienced by people with HCV infection, such as fatigue and mild cognitive impairment.^30^ In addition, individuals may experience stigma or discrimination associated with HCV infection.^31^ Individuals might also feel concern about their health—especially given that most of the study period fell before the introduction of direct-acting antivirals, at a time when HCV was more difficult to cure.

The effect size that we found was greater than that found in previous studies, particularly for those without liver cirrhosis. While most previous utility studies in HCV patients have lacked a non–HCV-infected control group, we identified only 5 studies that included a control group. Three of these studies focused on HCV infection in a general population sample^7–9^ while 2 focused on subpopulations of HCV patients that may not be comparable to our study population (people who inject drugs who were attending harm reduction services^32^ and blood donors^33^). The 3 general population studies found a utility decrement of 0.03 to 0.04^7–9^ associated with HCV infection compared with an uninfected control group. All 3 studies relied on self-reported HCV infection status and had low response rates. Our study used testing data to determine HCV status and relied on survey data with a higher response rate. This could suggest that the burden of hepatitis C was not fully captured in previous studies—for example, due to discrepancies between actual and self-reported hepatitis C infection status or bias introduced by low response rates. Some of the difference could also be explained by the different geographic regions represented by each study—for example, cultural differences in perception of health or differences in which subpopulations are most likely to receive hepatitis C testing.

We conducted a sensitivity analysis in which we further stratified our population by Ab, RNA, and testing status and examined their health utilities. We found that those who had HCV testing records had lower utilities than those with no testing records did (note: only those with testing records were included in the base-case analysis, in order to have a comparable control group). This is an expected finding as testing is likely to be focused on those who have risk factors for hepatitis C such as injection drug use, which is itself associated with a lower quality of life.^34^

Interestingly, the RNA-positive group and the Ab-positive RNA-negative group had similar health utilities. This may be due to a small sample size for the latter group (*n* = 62), or it could suggest that HCV antibody positivity is associated with a lower health utility (compared with those who are Ab negative), whereas viremia (RNA positivity) is not associated with an additional reduction in health utility (compared with those who are Ab positive and RNA negative). We also found that the Ab-positive RNA-untested group had lower health utilities than the Ab-positive RNA-negative group did. This is consistent with previous literature in which individuals with low health care engagement have been shown to have a low quality of life and health utility.^35^ This subgroup had the lowest quality of life of any subgroup analyzed in our study, which may point to a need to increase access to testing and care for the most vulnerable populations of hepatitis C patients who face barriers to accessing existing health services.

This study has the benefit of including a large sample designed to be representative of the general population of Ontario, including individuals in more remote or rural settings who are typically less likely to be captured in health utility studies. We also had access to a rich dataset that included health care administrative data and HCV testing data, whereas some previous studies have relied on self-reported HCV status and comorbidities. However, our study also has some limitations. We did not have access to HCV testing data for tests conducted outside of the Public Health Ontario Laboratory system, such as tests conducted at hospital and commercial labs. The PHOL database contains records for most HCV tests, especially RNA tests, that take place in Ontario, but the tests conducted elsewhere may be systematically different. In addition, we defined our HCV-positive cohort as those who ever tested positive for HCV. Some of these individuals were diagnosed with HCV after their CCHS survey date. We made this assumption in order to maximize our sample size, based on the fact that many individuals with HCV infection are unaware of their infection and are not diagnosed immediately. It is possible that some of these individuals were not yet infected with HCV at the time of CCHS survey completion. While the clinical and sociodemographic variables included in our analysis included many of the variables that have been identified in the literature as important predictors of HUI3 utilities in hepatitis C patients (sex, race, marital status, income, comorbidity, and mental health history), the analysis lacked a few key variables (education, employment status).^3,36–39^ Lastly, while our sample included a large number of low-income individuals, it is likely that some socioeconomically marginalized groups would not be represented in this study due to no or low interaction with the health care system and/or not being included in the CCHS. For example, the CCHS does not include First Nations peoples living on reserve and people experiencing homelessness^40^—both of which are groups that are disproportionately affected by hepatitis C.

## Conclusion

Overall, we showed that HCV is associated with reduced health utility in a large representative sample of Ontarians. Individuals with characteristics associated with socioeconomic marginalization had even lower health utilities, including those with a low household income and those who may experience barriers to accessing health care services such as HCV testing. In addition to improving access to HCV testing and treatment for these individuals, it may be beneficial to provide more social services such as mental health and financial supports to address comorbidities and social determinants of health to further improve their quality of life and health utility.

## Supplemental Material

sj-docx-1-mdm-10.1177_0272989X251319342 – Supplemental material for Health Utilities in People with Hepatitis C Virus Infection: A Study Using Real-World Population-Level Data Supplemental material, sj-docx-1-mdm-10.1177_0272989X251319342 for Health Utilities in People with Hepatitis C Virus Infection: A Study Using Real-World Population-Level Data by Yasmin A. Saeed, Nicholas Mitsakakis, Jordan J. Feld, Murray D. Krahn, Jeffrey C. Kwong and William W. L. Wong in Medical Decision Making
