# Supplementary material for: Health Utilities in People with Hepatitis C Virus Infection: A Study Using Real-World Population-Level Data
Source: Med Decis Making. 2025 Feb 22;45(3):332–43. doi: 10.1177/0272989X251319342 (PMC11894892; doi:10.1177/0272989X251319342)
Supplement: sj-docx-1-mdm-10.1177_0272989X251319342 – Supplemental material for Health Utilities in People with Hepatitis C Virus Infection: A Study Using Real-World Population-Level Data [file sj-docx-1-mdm-10.1177_0272989X251319342.docx]

# Appendices

## Appendix 1: Propensity score matching

##
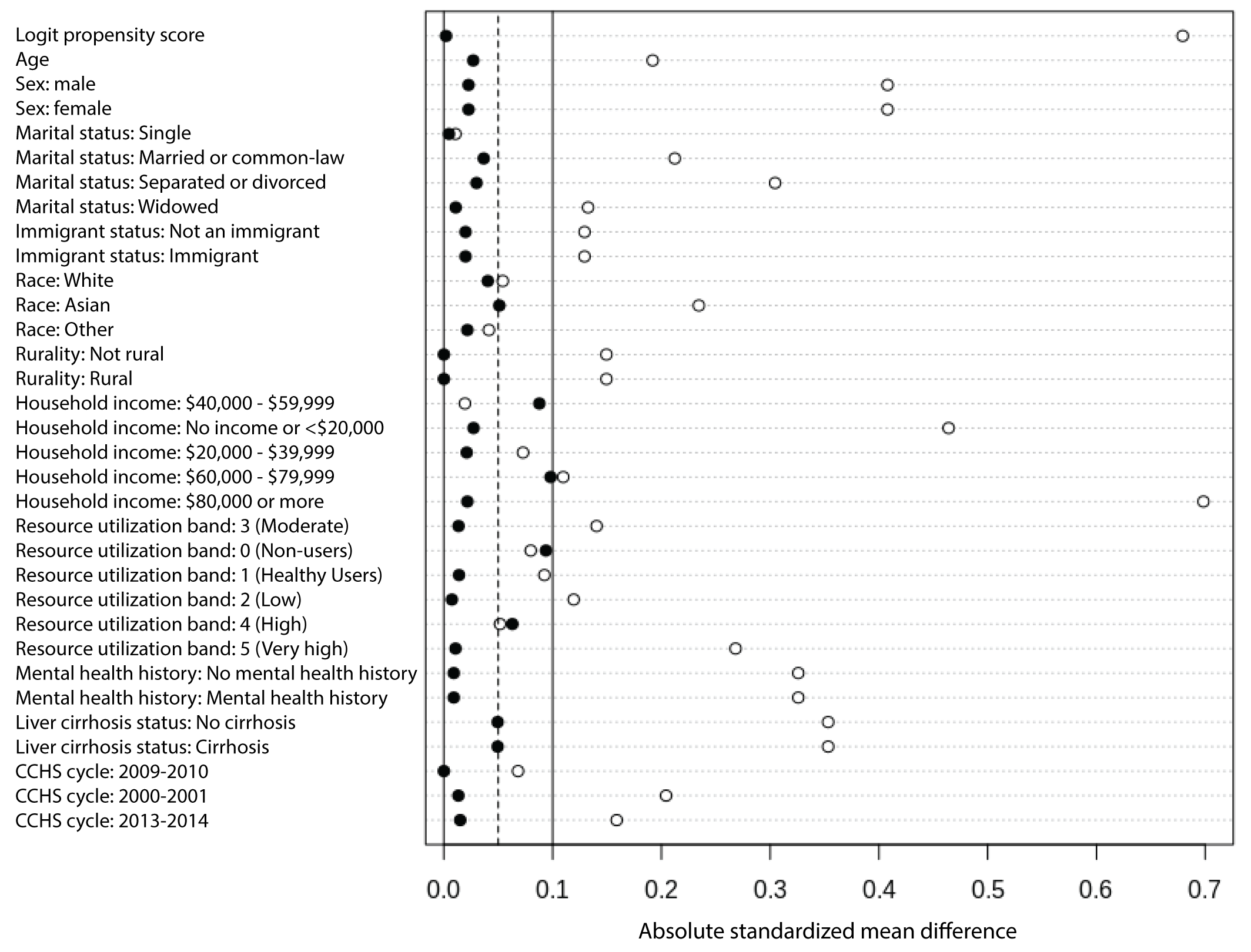
Table A1.1: Standardized mean differences before and after propensity score matching

Absolute standardized mean differences (exposed - control). White circles: unmatched data; black circles: matched data.

Standard deviation of all observations used to compute standardized differences.

## Appendix 2: Diagnostic codes used to identify cirrhosis

## Table A2.1: Diagnostic codes used to identify cirrhosis

| **Inpatient cirrhosis diagnostic codes** | **Inpatient diagnostic codes, ICD-9** | **Inpatient diagnostic codes, ICD-10** |
| --- | --- | --- |
| Toxic liver disease with fibrosis and cirrhosis of liver |  | K71.7 |
| Alcoholic cirrhosis of liver | 571.2 | K70.3 |
| Esophageal varices without bleeding | 456.1 | I85.9, I98.2 |
| Cirrhosis of liver without alcohol | 571.5 | K74.6 |

Searched the Canadian Institute for Health Information’s Discharge Abstract Database (DAD) and the National Ambulatory Care Reporting System (NACRS) database to identify cirrhosis cases.

Criteria: a single inpatient cirrhosis code from Table A2.1.

ICD-9: International Classification of Diseases, Ninth Revision; ICD-10: International Classification of Diseases, Tenth Revision.
